# Incidence and spatial variation of Parkinson's disease in the Netherlands (2017–2022): a population-based study

**DOI:** 10.1016/j.lanepe.2025.101565

**Published:** 2026-01-21

**Authors:** Mariana Simões, Susan Peters, Anke Huss, Sirwan K.L. Darweesh, Bastiaan R. Bloem, Roel Vermeulen

**Affiliations:** a Institute for Risk Assessment Sciences, Division of Environmental Epidemiology, Utrecht University, Utrecht, the Netherlands; b Radboud University Medical Centre, Donders Institute for Brain, Cognition and Behaviour, Department of Neurology, Center of Expertise for Parkinson and Movement Disorders, Nijmegen, the Netherlands; c Julius Center for Health Sciences and Primary Care, University Medical Center Utrecht, Utrecht, the Netherlands

**Keywords:** Parkinson's disease, Incidence, Mortality records, Health insurance data, Drugs prescription data, Hospital data, Spatial distribution

## Abstract

**Background:**

Parkinson's disease (PD) is a growing public health concern with a largely unknown aetiology. Understanding temporal trends, demographic drivers, and regional variations in PD risk is critical for guiding healthcare planning and identifying potential environmental and socioeconomic risk factors. We aimed to investigate the incidence and spatial distribution of PD in the Netherlands from 2017 to 2022, and examined demographic and socioeconomic disparities in PD risk.

**Methods:**

We conducted a nationwide, population-based study leveraging complementary strengths of multiple independent administrative health records linked to demographic and socioeconomic data. Incident PD cases were identified using a newly developed algorithm integrating mortality records, hospital data, health insurance claims, and medication prescriptions. We estimated overall and stratified age- and sex-internally standardized PD incidence rates (IRs). Spatial variations in PD risk were assessed using Bayesian hierarchical models to generate smoothed relative risk estimates at a fine-grained neighbourhood level.

**Findings:**

Between 2017 and 2022, we identified 22,343 incident PD cases in a population of 19,995,771 individuals (totalling 105,027,472 person-years at risk). Overall standardized IR was relatively stable across the evaluated time at 21.8 (95% confidence interval (CI): 21.6–22.1) per 100,000 person-years at risk. Incidence increased with age (peaking at 75–85 years) and was higher in men than women, in individuals with higher socioeconomic position, and residents of the northern provinces. Spatial analysis revealed significant geographic clustering of PD risk, which did not ecologically align with major environmental indicators such as air pollution, agricultural activity, or urbanization.

**Interpretation:**

The multifaceted algorithm offers a robust PD case ascertainment tool that allowed for a comprehensive nationwide assessment of PD incidence. In the Netherlands, this new approach uncovered regional disparities in PD risk that are not readily explained by known environmental indicators, warranting further investigation into potential environmental and socioeconomic determinants.

**Funding:**

Woelse Waard, Gieskes-Strijbis, and Ministry of Agriculture, Fisheries, Food Security and Nature of the Netherlands.

**Research in context**

**Evidence before this study**

Previous studies established the multifactorial aetiology of PD, involving both genetic and environmental factors. We searched PubMed for articles on nationwide incidence trends of Parkinson's disease (PD) published in English, Portuguese, Spanish, French, Italian, or Dutch until March 18, 2025. The search terms used were ‘Parkinson's disease’ AND ‘incidence’ AND (‘change’ OR ‘trend’ OR ‘variation’ OR ‘follow-up’) AND (‘population-based’ OR ‘registry OR 'registries' OR ‘nationwide’). While PD prevalence is rising globally—particularly in low- and middle-income countries—incidence rates remain underexplored, with substantial variation reported across countries. Population-based studies in high-income countries show differential risks for men vs women, age groups, co-morbidities, lifestyle, living in isolation vs in a community, socio-economic position and occupational and environmental exposures. However, differences in case ascertainment methods, population coverage, and statistical approaches hinder meaningful comparisons of incidence rates across countries. Moreover, only few studies have investigated geographical disparities in PD incidence. Rigorous case ascertainment and nationwide coverage are needed to accurately determine PD incidence and its geographical distribution, both of which are critical for etiological research aimed at identifying the primary drivers of PD within specific populations.

**Added value of this study**

Using a novel and comprehensive approach, we leveraged four nationwide health-related administrative data sources to developed a robust algorithm to reliably identify incidence PD cases in the Netherlands. The algorithm incorporates both the likelihood of a true PD diagnosis and the certainty of diagnosis date, generating a confidence score that reflects these two dimensions. Combining the strengths of each source enhanced the rigor of the case ascertainment. Integrating this information with other registers with individual-level information, we computed, for the first time, nationwide overall sex- and age-standardized incidence rates (IRs), and stratified these rates by demographic, socio-economic and area-level characteristics. Spatial analysis revealed distinct geographic clustering of PD incidence at a fine-grained neighbourhood level, suggesting a possible environmental contribution to PD. Notably, these patterns could not readily be explained, on an ecological level, by known environmental indicators such as air pollution, agricultural activity, or urbanization. These findings highlighting the need for further investigation into local factors that may influence PD risk. In line with previous findings from other high-income countries, we observed higher PD incidence in men and in older age groups.

**Implications of all the available evidence**

Given the face-validity of our approach to identify incident PD cases, our methodology provides a robust foundation for future individual-level analyses focused on the environmental, genetic, and socioeconomic drivers of PD. Such analyses have the potential to generate critical insights and guide targeted public health interventions aimed at modifiable risk factors.

## Introduction

Parkinson's disease (PD) is the second most common neurodegenerative disorder, with prevalence rising globally, driven in part by population aging and likely influenced by environmental and other factors.^1^^,^^2^ PD is associated with progressive disability, higher risk of dementia and mortality, and imposes substantial economic and public health burdens.^3^

While the global prevalence of PD is increasing, incidence trends remain less well characterized, with inconsistent results across studies. Some investigations report rising incidence, whereas others suggest stable, or even declining trends. A study in North Korea reported an increase in PD incidence between 2010 and 2015,^4^ while Liu et al. noted an increase between 2002 and 2009 in Taiwan.^5^ However, another study in Taiwan covering an earlier period (2005–2011) showed a decrease in PD incidence.^6^ In the United States, Savica and colleagues observed an increase in PD incidence over a period of 30 years (1976–2005)^7^; in contrast, Akushevich and colleagues observed a stable trend between 1992 and 2005.^8^ In Europe, the results from studies in the Netherlands, Germany and in the United Kingdom suggested a declining trend in the incidence of PD, in 1990–2000, in 2004–2009 vs 2014–2019, and 1999–2009, respectively.^9^, ^10^, ^11^ Studies in Norway (between 2004 and 2017) and France (between 2002 and 2018), however, suggested stable incidences.^12^^,^^13^ These inconsistencies may reflect differences in environmental exposures, genetic background, healthcare access, or data quality.^2^^,^^14^, ^15^, ^16^ Notably, recent research suggests that rural residency, agricultural pesticide exposure and air pollution may contribute to regional differences in PD risk.^2^^,^^16^^,^^17^

PD incidence has been associated with sociodemographic characteristics, including age, sex, and socioeconomic position.^2^ While it is well established that PD incidence increases with age and is higher in men, the role of socioeconomic factors remains debated. Some evidence suggests an earlier diagnosis in higher-income groups due to better healthcare access, while other studies point to higher exposure to risk factors and healthcare disparities in lower-income populations.^14^^,^^18^ Given the growing burden of PD and the potential for modifiable risk factors, a detailed understanding of incidence patterns across sociodemographic groups is essential.

This study aims to provide the first comprehensive nationwide assessment of PD incidence in the Netherlands from 2017 to 2022. We estimate overall and stratified age- and sex-standardized incidence rates, and assess spatial distribution of PD and its relationship with regional and socioeconomic characteristics. By leveraging administrative health data from multiple independent sources and advanced spatial epidemiological methods, this study aims to generate novel insights into the spatial and demographic patterns of PD risk, with potential implications for healthcare policy and disease prevention strategies.

## Methods

### Data sources and PD ascertainment

PD cases were identified using a novel approach using complementary strengths of four independent data sources: mortality registry data, health insurance data and electronic medical records, and medication prescription data.

1. Mortality registry

The mortality registry contains data on the main cause of death of all deceased residents of the Netherlands from 1995 to 2023. Causes of death are coded using the International Classification of Diseases and related Health Problems 10th edition (ICD-10), of which “G20” corresponds to PD. While highly specific,^19^ using mortality data alone substantially underestimates PD cases, and death dates do not reflect actual diagnosis dates.^2^

2. Health insurance data (named Vektis)

Vektis health insurance claims data provide a near-complete overview of secondary care services in the Netherlands, covering over 99% of all consultations, diagnoses, treatments, and hospitalizations. We used Vektis records from 2016 to 2023, and identified PD diagnoses using the specialism + diagnosis code 0330–0501, indicating a neurologist-confirmed diagnosis of PD. Despite high diagnostic certainty, data truncation at 2016 poses challenges distinguishing between incident and prevalent cases.

3. Electronical hospital data (Dutch Hospital data, DHD)^20^

Two national hospital databases were used: the National Medical Registration (1995–2012, ICD-9: 332.0) and the National Basic Hospital Care Register (2013–2022, ICD-10: G20). Both include all hospitalizations where PD was the underlying diagnosis. Hospital data have high diagnostic certainty but do not necessarily reflect the year of diagnosis, as PD-related hospital visits may be linked to treatment, disease progression or co-morbidities rather than initial diagnosis.

4. Medication prescription data

Information on medication prescribed by doctors, dispensed through pharmacies and registered in the basic health insurance package was available from 2006 to 2023. Prescribed medication, classified as Anatomical Therapeutic Chemical (ATC) codes up to 4th level, are recorded as one entry per year per medication type. We identified subjects that used anti-Parkinson drugs: anticholinergic agents (N04A), dopaminergic agents (N04B) and other anti-Parkinson drugs (N04C). ATC codes refer to the main indication for which the drug is registered. Since these drugs can be prescribed for other health problems (e.g., N04B for restless legs) in isolation they are not reliable to identify PD cases. Nonetheless, in the Netherlands, most PD patients begin anti-Parkinson treatment within the first year following diagnosis, making prescription records a suitable indicator for determining the timing of PD diagnosis.

**Fig. 1:**
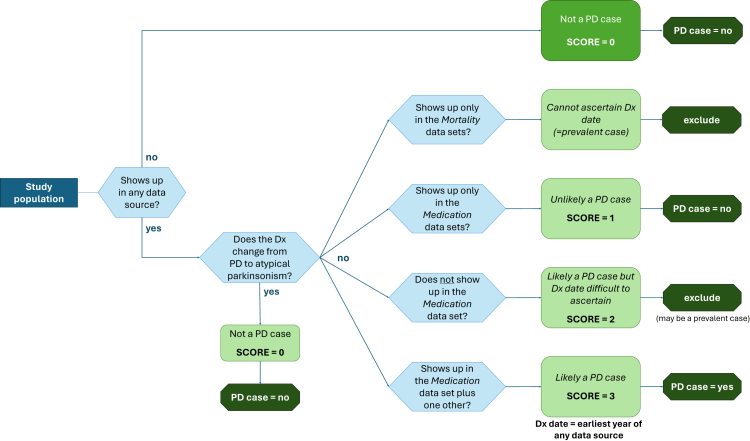
**Algorithm for identifying incident Parkinson's disease (PD) cases using administrative datasets in the Netherlands**.

We developed an innovative algorithm (Fig. 1) that integrates these four data sources to identify incident PD cases, assigning a confidence score based on the likelihood of a true PD diagnosis and the certainty of diagnosis date.

• Score 0 (Not PD cases): Individuals with no recorded PD-related events in any data source were classified as non-cases. Additionally, those initially diagnosed with PD but later reclassified as having atypical parkinsonism (n = 987) were also considered non-cases.
• Score 1 (Likely non-PD cases): Individuals prescribed anti-Parkinson medication but without an entry in the mortality, Vektis, or DHD datasets were considered unlikely to have PD, as medications may be prescribed for other purposes.
• Score 2 (Possibly prevalent PD cases): Individuals identified as PD cases in the mortality, Vektis, or DHD databases but without anti-Parkinson medication records were classified as PD cases. However, since the timing of diagnosis could not be determined with confidence, they were excluded from incidence analyses to prevent inclusion of prevalent cases.
• Score 3 (Incident PD cases): Individuals with entries in at least one of the high-certainty PD data sources (mortality, Vektis, or DHD) who also had a prescription for anti-Parkinson medication were classified as incident PD cases. The diagnosis year was assigned as the earliest year in which they appeared in any of the data sources.

### Study population

**Fig. 2:**
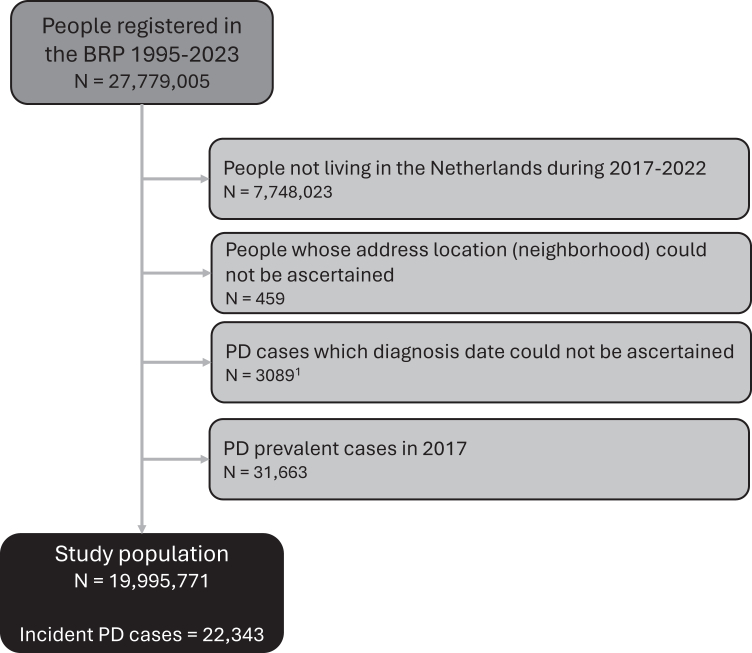
**Flow chart of the study population.** BRP, Personal Records Database (Basisregistratie Personen in Dutch). ^1^ PD cases only in the mortality registry (n = 844) and PD cases with score 2 (n = 2245).

From the population register (Personal Records Database, BRP) of Statistics Netherlands, we selected all persons that resided in the Netherlands between 2017 and 2022 (Fig. 2). The first recorded residential address during this period was used to assign individuals to a municipality and neighbourhood. Individuals whose address could not be determined were excluded from the study.

To identify incident PD cases, we applied the case ascertainment algorithm described above. Prevalent cases, defined as individuals with a PD score of 3 and a diagnosis year before 2017, were excluded ensuring that only newly diagnosed cases were included in the incidence analysis. Individuals for whom a reliable diagnosis date could not be determined were excluded (those who appeared only in the mortality registry or had a PD score of 2).

Data include information pertaining to demographic, socio-economic and neighbourhood characteristics: sex, age, migration background, living with a partner, household income, household socio-economic position, neighbourhood's socio-economic position, neighbourhood's urbanization degree and province. All individual-level data (microdata) were linkable at the individual level via a unique pseudonymised identifier (Table S1 and Figure S1).

### Statistical analysis

#### PD incidence rates and incidence risk ratios

Sex and age internally standardized incidence rates (IRs) of PD were calculated per year and for the entire study period (2017–2022) and reported per 100,000 person-years at risk (PYAR). The 2019 Dutch population was used as the reference for standardization. Standardized IRs were also computed for several strata based on the individual and area level characteristics as listed in the previous section. We used negative binomial regression to obtain incidence risk ratios (IRRs) comparing each year with the previous year and for the study period (2022 vs 2017). Data were aggregated by sex and 10-year age-groups and models were adjusted for number of residents of Dutch origin (to ensure that demographic changes in population origin over time do not affect the standardized numbers), number of persons living with a partner, number of persons in 5th quintile of household income and number of persons in the 5th quintile of household socio-economic position score in each group. The log of time-at-risk was used as offset.

#### PD incidence relative risk—spatial mapping and cluster analysis

To evaluate the geographic distribution of PD incidence, we calculated the standardized incidence ratio (SIR), as the ratio of observed to expected cases, for each of the 2980 neighbourhoods in the Netherlands. Expected number of PD cases per neighbourhood was determined using a reference population stratified by sex and 5-year age groups. Because SIRs can be unreliable in small populations, and due to disclosure restrictions (CBS regulation) prohibiting reporting of estimates based on fewer than 10 observations, we applied a Bayesian hierarchical model to generate smoothed relative risks (RR). The model uses integrated nested Laplace approximation (INLA) as the method to compute fast approximate posterior marginal distributions (further details in Textbox S1). The output is the estimated sex and age-group standardized RR of PD per neighbourhood, compared to the whole country, and their 95% credible intervals. Estimated RRs were used to map PD incidence in the Netherlands for 2017–2022. To measure spatial autocorrelation, we computed Global Moran's I statistic and its corresponding P value based on the estimated RRs and their credible intervals. We used queen contiguity-based spatial weights to encode the spatial relationship between neighbourhoods.^21^ We further applied a cost to the edges of the neighbourhoods based on Euclidean distance of their centroids to account for the high variability in neighbourhood size. Moran's I evaluates whether an attribute of a spatial feature (neighbourhood in this case) has a dispersed, random or clustered pattern, reflected by a statistically significant negative, a non-significant, or statistically significant positive Global Moran's I, respectively.

Furthermore, we performed a cluster analysis using a spatially constrained clustering method to assure that the neighbourhoods were spatially contiguous—the Spatial C (K)luster Analysis by Tree Edge Removal (SKATER) algorithm.^22^ This algorithm is based on pruning a minimum spanning tree (an acyclic connected tree with neighbourhoods as nodes) in a way that reflects the contiguity structure of the neighbourhoods while the cuts form clusters in which the neighbourhoods’ RRs and their credible intervals were as similar as possible.

#### Sensitivity analyses

We performed the following pre-planned sensitivity analyses for the overall IR:

(1) Restriction to the pre-pandemic period: As COVID-19 impacted the healthcare system resulting in diagnosis delays of several diseases, including PD, we restricted the analysis to 2017–2019. Additionally, by excluding PD cases diagnosed after 2019, we assured a minimum follow-up of 3 years after diagnosis, significantly reducing the chance that individuals whose initially diagnosis was PD could have been later reclassified as having atypical parkinsonism (highest conversion rates occur within the first year after PD diagnosis, rarely converting after three years)
(2) Censoring individuals upon entry into a nursing home or mental health institution: In the Netherlands, individuals residing in nursing homes or mental health institutions are typically not followed up in hospital records, which could introduce bias in case ascertainment. To examine the impact of this, we performed an analysis where follow-up was terminated when individuals transitioned into these facilities.
(3) Additional stratified analyses: To further investigate specific subgroups, we computed IRs for combinations of selected characteristics, specifically: sex and migration background, sex and province, and age and province. Furthermore, RRs stratified by sex and by age (<60, 60–80 and > 80 years of age) were estimated and mapped.

Additionally, sensitivity analyses using different spatial weights for computing the global Moran's I were performed (contiguity without application of cost of edges, Euclidean distance, and k-nearest neighbour, Table S2).

#### Software

All analyses were carried out in R 4.4.0,^23^ using the sf,^24^ SpatialEpi,^25^ spdep,^24^ INLA,^26^ MASS,^27^ and performance R packages.^28^

### Ethics statement

This study required no separate approval from an Ethics Committee nor individual informed consent. Analyses were performed on non-identifiable microdata provided under Statistics Netherlands (CBS) Microdata Services and accessed in the secure CBS microdata environment in accordance with CBS access conditions and applicable privacy legislation.

### Role of the funding source

The funders of the study had no involvement in the design, data collection, analysis, interpretation, or preparation of the manuscript. The first author had full access to all the data in the study. The corresponding author had full access to all the data in the study and had final responsibility for the decision to submit.

## Results

### Study population characteristics

The study population included 19,995,771 individuals residing in the Netherlands between 2017 and 2022, contributing to a total of 105,027,472 PYAR. During this period, 22,343 incident PD cases (score 3) were identified (Table 1; number of individuals per score in Table S3). The median age at diagnosis was 73 years, most cases were male (14,305, 64.1%) and of Dutch origin (19,158, 85.8%).

**Table 1:** Characteristics of the study population.

Characteristic	Without PD	With incident PD
**N**	19,534,762	22,343
**Age**		
Median [IQR]	40.00 [21.00, 60.00]	73.00 [67.00, 79.00]^a^
**Sex**		
Male [n (%)]	9,725,910 (49.8)	14,305 (64.0)
Female [n (%)]	9,808,852 (50.2)	8038 (36.0)
**Migration background**^b^		
The Netherlands [n (%)]	1,4016,267 (71.8)	19,158 (85.7)
Morocco [n (%)]	442,372 (2.3)	289 (1.3)
Turkey [n (%)]	476,520 (2.4)	209 (0.9)
Suriname [n (%)]	381,715 (2.0)	228 (1.0)
Former Dutch Antilles and Aruba [n (%)]	198,981 (1.0)	86 (0.4)
Other non-Western countries [n (%)]	1,408,518 (7.2)	340 (1.5)
Other Western countries [n (%)]	2,610,232 (13.4)	2033 (9.1)
Unknown [n (%)]	157 (0.0)	0 (0.0)
**Living with partner**		
No [n (%)]	10,588,531 (54.2)	5604 (25.1)
Yes [n (%)]	8,946,231 (45.8)	16,739 (74.9)
**Household income percentile**		
Median [IQR]	64.00 [38.00, 84.00]	53.00 [34.00, 73.00]
1st quintile (lowest income) [n (%)]	3,521,550 (18.0)	4653 (20.8)
2nd quintile [n (%)]	3,573,273 (18.3)	7271 (32.5)
3rd quintile [n (%)]	3,341,109 (17.1)	4584 (20.5)
4th quintile [n (%)]	3,510,397 (18.0)	2987 (13.4)
5th quintile (highest income) [n (%)]	3,434,628 (17.6)	2635 (11.8)
Unknown [n (%)]	2,153,805 (11.0)	213 (1.0)
**Household SEP score**^c^		
Median [IQR]	5.27 [−2.85, 9.70]	1.79 [−4.05, 6.63]
1st quintile (lowest SEP) [n (%)]	3,359,135 (17.2)	4480 (20.1)
2nd quintile [n (%)]	3,288,983 (16.8)	8132 (36.4)
3rd quintile [n (%)]	3,330,817 (17.1)	4396 (19.7)
4th quintile [n (%)]	3,335,283 (17.1)	2949 (13.2)
5th quintile (highest SEP) [n (%)]	3,336,868 (17.1)	1966 (8.8)
Unknown [n (%)]	2,883,676 (14.8)	420 (1.9)
**Neighborhood's SEP score**^c^		
Median [IQR]	0.03 [−0.17, 0.16]	0.05 [−0.12, 0.17]
1st quintile (lowest SEP) [n (%)]	3,510,424 (18.0)	2996 (13.4)
2nd quintile [n (%)]	3,560,474 (18.2)	4066 (18.2)
3rd quintile [n (%)]	3,528,009 (18.1)	4319 (19.3)
4th quintile [n (%)]	3,522,107 (18.0)	4262 (19.1)
5th quintile (highest SEP) [n (%)]	3,504,557 (17.9)	4334 (19.4)
Unknown [n (%)]	1,909,191 (9.8)	2366 (10.6)
**Neighborhood's urbanization degree**		
Extremely urbanized (>2500 addresses per km^2^) [n (%)]	4,903,594 (25.1)	4494 (20.1)
Strongly urbanized (1500–2500 addresses per km^2^) [n (%)]	5,415,973 (27.7)	6326 (28.3)
Moderately urbanized (1000–1500 addresses per km^2^) [n (%)]	3,115,632 (16.0)	3710 (16.6)
Hardly urbanized (500–1000 addresses per km^2^) [n (%)]	3,015,967 (15.4)	3891 (17.4)
Not urbanized (<500 addresses per km^2^) [n (%)]	3,078,065 (15.8)	3917 (17.5)
Unknown [n (%)]	5531 (0.0)	5 (0.0)
**PD diagnosis year**		
2017 [n (%)]	–	3726 (16.7)
2018 [n (%)]	–	3869 (17.3)
2019 [n (%)]	–	3837 (17.2)
2020 [n (%)]	–	3455 (15.5)
2021 [n (%)]	–	3787 (16.9)
2022 [n (%)]	–	3669 (16.4)

IQR, interquartile range; SEP, socioeconomic position.

a Age at PD diagnosis.

b Defined by Statistics Netherlands, where persons whose parents were both born in the Netherlands have a Dutch background and persons with migration background are either assigned the country they were born in (person with first-generation migration background) or the country their mother was born in (or, in case the mother was born in the Netherlands, the country where the father was born in; person with second-generation migration background).

c SEP standardized scores were computed by CBS based on financial wealth, education level and recent employment history of households.

### Incidence rates and incidence risk ratios

**Fig. 3:**
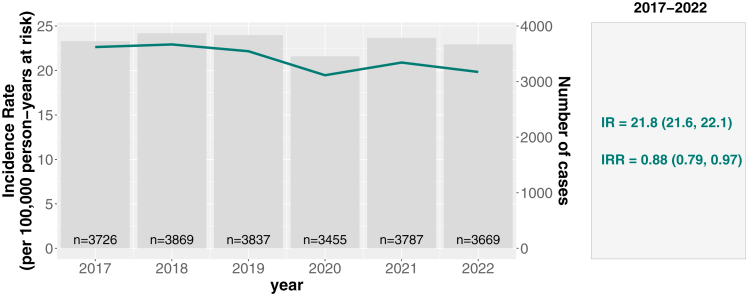
**Parkinson's disease (PD) in the Netherlands in 2017–2022. Left: annual incident cases (bars) and sex and 5-year age group standardized incidence rates (IR; line). Right: overall IR for 2017–2022 and IRR for 2022 vs. 2017, with 95% confidence intervals**.

The sex- and age internally standardized incidence rate (IR) of PD for the period 2017–2022 was 21.8 (95% confidence interval (CI): 21.6–22.1) per 100,000 person-years at risk (PYAR). A small gradual decline was observed over time, with the IR decreasing from 22.6 (21.9, 23.3) per 100,000 PYAR in 2017 to 19.8 (19.2, 20.5) per 100,000 PYAR in 2022, representing a 12% relative reduction (Fig. 3, Table S4). This trend corresponded to an incidence risk ratio (IRR) of 0.88 (95% CI: 0.79–0.97) when comparing 2022 to 2017.

The year-over-year number of cases, IRs and IRRs were essentially stable between 2017 and 2019 followed by a decline in 2020 coinciding with the onset of the COVID-19 pandemic. Sensitivity analyses confirmed the robustness of the estimated incidence rates, with a sex- and age-standardized IR of 22.5 (22.1, 22.9) per 100,000 PYAR when restricting the analysis to 2017–2019 and 22.7 (22.5, 23.0) per 100,000 PYAR after excluding individuals residing in nursing homes.

The standardized IR of PD was higher in men than in women and increased with age, particularly in individuals over 70 years of age, except for those over 90 years (Table 2). The male-to-female ratio was approximately 1.81 (IRR = 1.90 (95% CI: 1.70–2.20)). A slightly lower disparity in older age groups (Table S5) was observed. However, fluctuations in case numbers at older ages introduce some uncertainty to this estimate.

**Table 2:** Standardized incidence rates (IRs) of Parkinson's disease by individual and area-level characteristics in 2017–2022.

Stratum	Numerator	Denominator	Incidence rate (95% CI) per 100,000 person-years at risk
**Overall**^a^	22,343	105,027,472.2	21.8 (21.6, 22.1)
**Age**^b^			
<40	74	49,501,474.27	0.1 (0.1, 0.2)
40–50	382	13,387,406.3	2.9 (2.6, 3.1)
51–60	1848	15,272,959.89	12.1 (11.5, 12.6)
61–70	5077	12,662,733.96	40.1 (39.0, 41.2)
71–80	9528	9,334,670.34	102.2 (100.1, 104.2)
81–90	5133	4,061,624.222	126.6 (123.1, 130.1)
>90	301	806,603.2685	37.3 (33.1, 41.5)
**Sex**^c^			
Male	14,305	52,223,904.84	28.2 (27.7, 28.7)
Female	8038	52,803,567.4	15.6 (15.2, 15.9)
**Migration background**^a^			
The Netherlands	19,158	79,204,362.68	22.0 (21.7, 22.4)
Morocco	289	2,477,343.159	30.0 (27.9, 32.2)
Turkey	209	2,548,659.912	23.2 (21.3, 25.1)
Suriname	228	2,174,130.096	19.3 (17.4, 21.1)
Former Dutch Antilles and Aruba	86	1,052,490.151	22.8 (20.0, 25.7)
Other non-Western countries	340	6,376,100.638	20.0 (18.9, 21.1)
Other Western countries	2033	11,194,274.02	20.5 (19.6, 21.3)
**Living with partner**^a^			
No	5604	53,105,800.94	18.7 (18.3, 19.0)
Yes	16,739	51,921,671.31	23.4 (23.0, 23.8)
**Household income percentile**^a^			
1st quintile (lowest income)	4653	19,011,477.25	18.1 (17.5, 18.7)
2nd quintile	7271	20,505,650.62	21.6 (21.0, 22.3)
3rd quintile	4584	19,598,839.81	24.0 (23.3, 24.7)
4th quintile	2987	20,769,652.59	25.2 (24.5, 25.8)
5th quintile (highest income)	2635	20,319,015.56	25.0 (24.4, 25.7)
Unknown	213	4,822,836.4	38.7 (37.0, 40.5)
**Household SEP score**^a^			
1st quintile (lowest SEP)	4480	18,796,659.87	18.5 (17.9, 19.1)
2nd quintile	8132	18,746,277.04	21.9 (21.2, 22.5)
3rd quintile	4396	19,595,071.28	24.3 (23.7, 25.0)
4th quintile	2949	19,757,809.55	25.0 (24.3, 25.7)
5th quintile (highest SEP)	1966	19,807,846.89	26.1 (25.4, 26.8)
unknown	420	8,323,807.605	19.7 (18.8, 20.7)
**Neighborhood's SEP score**^a^			
1st quintile (lowest SEP)	2996	18,117,278.36	19.9 (19.2, 20.5)
2nd quintile	4066	19,128,482.18	21.6 (21.0, 22.3)
3rd quintile	4319	19,242,814.15	22.0 (21.4, 22.7)
4th quintile	4262	19,226,257.78	21.9 (21.2, 22.6)
5th quintile (highest SEP)	4334	19,083,381.39	22.8 (22.1, 23.4)
Unknown	2366	10,229,258.39	23.0 (22.0, 23.9)
**Neighborhood's urbanization degree**^a^			
Extremely urbanized (>2500 addresses per km^2^)	4494	25,050,615.5	21.9 (21.3, 22.5)
Strongly urbanized (1500–2500 addresses per km^2^)	6326	29,402,904.83	21.9 (21.3, 22.4)
Moderately urbanized (1000–1500 addresses per km^2^)	3710	17,217,145.35	21.6 (20.9, 22.3)
Hardly urbanized (500–1000 addresses per km^2^)	3891	16,489,056.1	22.1 (21.4, 22.8)
Not urbanized (<500 addresses per km^2^)	3917	16,836,002.13	21.8 (21.1, 22.5)

CI, confidence interval; SEP, socioeconomic position.

a Sex and 5-year age groups standardized.

b Sex standardized.

c 5-year age groups standardized.

Comparisons across individual and area-level characteristics showed similar age- and sex-standardized IRs among different ethnic groups. Individuals living with a partner had higher IRs compared to those without a partner (Table 2). Individuals from lower-income households and lower household socioeconomic position (SEP) groups had lower IRs. Consistently, IRs were lower in neighbourhoods with lower SEP. No clear differences in IRs were observed across neighbourhoods with different degrees of urbanization.

**Fig. 4:**
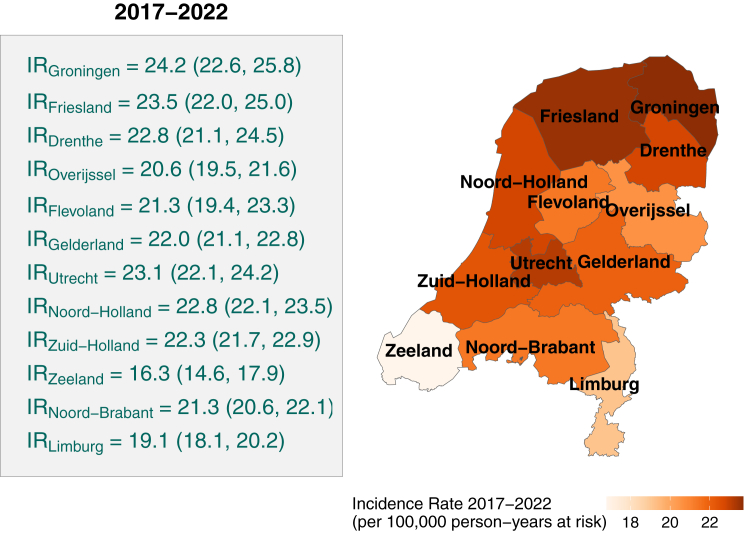
**Incidence rate of Parkinson's disease by province (2017–2022), adjusted for sex and 5-year age groups. Left: estimated incidence rates (with 95% confidence intervals). Right: geographic distribution of the same incidence rates, represented on a choropleth map**.

The geographic distribution of PD incidence is presented in the right panel of Fig. 4, which displays the overall IRs for 2017–2022 for each of the 12 administrative provinces in The Netherlands. The highest IRs were observed in the northern provinces of Friesland and Groningen, while the central and central-western provinces had rates in line with the national average. Lowest IRs were recorded in Zeeland and Limburg, in the south of the country. Additional analyses are provided in Tables S6–S9.

### Spatial distribution of PD

**Fig. 5:**
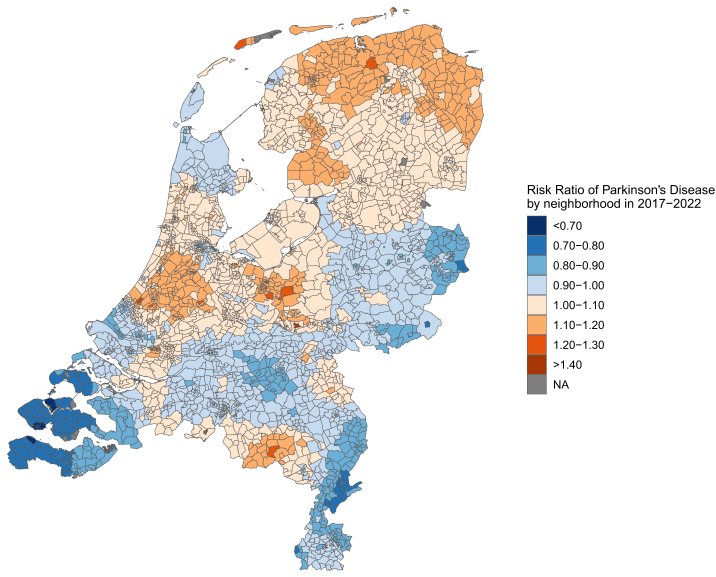
**Risk ratios of Parkinson's disease by neighbourhood in the Netherlands (2017–2022)**.

The relative risks (RRs) of PD across Dutch neighbourhoods in 2017–2022 varied by a factor of 2 from 0.66 (95% CI: 0.50–0.84) to 1.41 (1.13–1.75) (Fig. 5). This fine-grained, neighbourhood-level analysis provides a more detailed perspective on spatial variation than the broader provincial patterns shown in the right panel of Fig. 4, highlighting elevated RRs in the northern, central, and centre-western regions of the Netherlands. These spatial patterns were consistent across stratified analyses by sex and age (Figure S2).

Spatial autocorrelation analysis yielded a Global Moran's I of 0.83 (p < 0.0001), indicating a significant clustered distribution of PD incidence, with neighbourhoods exhibiting similarly high or low RRs tending to be geographically proximate.

Neighbourhood-level RR mapping also revealed a clustering pattern with regional differences in PD risk that exhibit consistently higher or lower IR than expected based on random spatial processes (Figure S3).

## Discussion

We leveraged multiple complementary administrative health records to estimate nationwide age- and sex-standardized incidence rates of Parkinson's disease (PD) in the Netherlands (2017–2022). Our findings indicate a relatively stable overall IR of 21.8 per 100,000 person-years at risk (PYAR), with higher rates among men, older adults, and individuals from higher socioeconomic backgrounds.

A key new finding was the presence of distinct geographic clustering of PD incidence, suggesting a possible environmental contribution to PD. However, this could not readily be explained, on an ecological level, by known environmental indicators such as air pollution, agricultural activity, or urbanization. Geographic clusters have been observed elsewhere: in France, areas with intensive vineyard cultivation, known for high pesticide use, were associated with elevated PD incidence.^29^^,^^30^ In Québec, Canada, persistent regional differences in PD prevalence have been reported, consistent with earlier ecological observations, reinforcing the hypothesis that environmental factors may contribute to spatial clustering of PD risk.^31^ These factors have been reported to contribute to PD by a variety of pathophysiological mechanisms, inducing mitochondrial and lysosome dysfunction, oxidative stress, and inflammation. Other pathophysiological mechanisms relate to promotion of α-synuclein misfolding and aggregation and impairment of protein degradation pathways. Toxic insults early on in the disease process may trigger pathological changes in either the gut (possibly following oral exposure to toxic chemicals) and olfactory system (possibly following inhalation of toxic chemicals) that subsequently lead to brain pathology.^32^^,^^33^ Additionally, both gene–environment interactions and advanced age can further increase susceptibility to the toxic effects of these environmental exposures.^33^^,^^34^

Our current analysis did not integrate quantitative environmental exposure models, as the primary aim of this work was to develop a reliable approach for ascertaining incident PD cases and to describe their socio-economic and spatial distributions across the Netherlands. This foundational step is necessary to ensure the robustness of case identification and spatial referencing before incorporating complex environmental data. To this aim, we combined four independent and routinely collected, high-quality health-related administrative databases, ensuring robustness and completeness in PD case ascertainment. We used mortality records, health insurance data where PD was registered under medical specialty (neurologists), anti-Parkinson drugs prescription data, and hospital data (in and out-patient). All data sources offered nationwide coverage and near-complete representation of the Dutch population. Prior studies typically relied on one or two data streams.^19^^,^^35^ In contrast, our multi-source approach strengthens confidence in our incidence estimates. Indeed, we observed known PD patterns, such as higher rates in older individuals and in men, supporting the face validity of our methodology.

Using this approach, we evaluated the spatial distribution of PD incidence in the Netherlands using an advanced spatial hierarchical model and created, for the first time, a fine-grained, neighbourhood-level incidence map. This revealed distinct geographic clusters of PD that were not readily explained by known environmental indicators. Unlike some studies reporting urban–rural differences in PD incidence,^5^^,^^9^^,^^11^ we found no such trend—consistent with findings from Latin America and Australia.^17^^,^^36^ Regionally, PD incidence was highest in the northern provinces (Groningen and Friesland) and lowest in the southern provinces (Limburg and Zeeland), forming a northeast-to-southwest gradient.

Understanding these spatial patterns may help identify modifiable environmental or lifestyle factors. However, as in previous studies,^14^^,^^15^ the observed regional disparities were not easily explained by ecological alignment with known environmental risk factors. Genetic predisposition, environmental exposures, lifestyle factors, healthcare access, and diagnostic practices may all contribute to these patterns. Yet, the widespread availability of PD-specialized centers in the Netherlands reduces the likelihood of major diagnostic disparities across regions. Although ecological indicators were not aligned with PD clustering, unmeasured or more localized environmental exposures may still play a role. A better understanding of the environmental contribution to PD requires a coupling of the present incidence data to a detailed mapping of the exposome. The latter approach was not part of this present study, but is currently being explored in ongoing work in The Netherlands.

In agreement with other studies,^4^^,^^5^^,^^9^^,^^12^^,^^14^^,^^15^ PD incidence increased with age, peaking at 75–85 years, before declining in those >90 years. The median age at diagnosis (73 years) aligns with findings from other population-based studies,^12^^,^^13^ while contrasting with the age distribution of patients in clinic-based studies, which tend to underrepresent older patients relative to the full PD patient population.

In line with previous research, we observed higher IRs in men (28.2 per 100,000 PYAR) compared to women (15.6 per 100,000 PYAR). IRs were in line with those reported in Norway (29.6 and 23.1 per 100,000 PY for men and women, respectively)^12^ but lower than in the UK (73.18 and 42.66 per 100,000 PY, respectively).^14^ The observed 1.8 male-to-female ratio was slightly higher than ratios observed in other studies (1.4–1.6).^4^^,^^12^^,^^37^ The ratio increased with age, similar to what was reported in the Norwegian and French studies.^12^^,^^37^ Variation in male-to-female ratios reported across studies can be explained by methodologic differences such as study design, case definition or diagnostic criteria. For example, there are more women than men in higher age categories (>70 years), which harbour the majority of incident PD cases. The underrepresentation of older PD cases in registry-based studies can thus lead to an overestimate of the unadjusted male-to-female ratio. Differences in male-to-female ratios can also arise from *bona fide* differences between countries in what concerns differential prevalence of potential PD risk/protective factors for PD in men and women, such as environmental, occupational and behavioural (lifestyle) determinants.

PD incidence was largely similar across migration background groups, also after stratification by sex. An exception was observed among individuals with Moroccan ancestry, who had a notably higher incidence rate and the lowest male-to-female ratio (1.4). Previous studies have reported ethnic disparities in PD risk,^2^ often attributed to genetic, occupational, or environmental factors, as well as differences in healthcare access. The framework of this study, where genetic data and detailed occupational information are unavailable, precludes further investigation on these putative risk factors. However, it is noteworthy to mention that Morocco has a high prevalence of several PD-associated genetic variants, including the highest known prevalence of the Gly2019Ser mutation in the *LRRK2* gene.^38^^,^^39^ Concomitantly, there is no evidence that people of Moroccan background have better access to healthcare in the Netherlands. On the contrary, there are concerns that people from non-Western migration minority groups may more often face barriers to obtaining a timely diagnosis of PD. Genetic, occupational and socioeconomic risk factors are better evaluated in more detailed studies (i.e. cohorts, case–control). PD incidence was higher among individuals living with a partner, possibly reflecting better case detection through partner support.^40^ Although more PD cases, in absolute terms, resided in lower income and socioeconomic position (SEP) households, higher standardized IRs were observed in individuals from wealthier households and in both household and neighbourhood higher-SEP strata. Although previous research has reported higher PD incidence in less deprived groups, findings have been inconsistent.^2^^,^^11^^,^^14^ Possible explanations include greater healthcare access, case detection, and differences in environmental exposures. Both differential detection and true differences in underlying risk factors (e.g., environmental or lifestyle exposures) may contribute to the observed socioeconomic gradient in the Netherlands. However, the Dutch healthcare system provides universal coverage with relatively equal access to general and specialist care. A study by Loef et al. showed that lower SEP groups incur larger healthcare utilization and expenditure, largely due to greater morbidity rather than better access among higher-SEP individuals.^41^ Smoking is particularly relevant in this socioeconomic context, as it is robustly associated with a lower PD risk, although the causal direction remains uncertain. Smoking behaviour is less prevalent in higher SEP individuals in the Netherlands,^42^ which may contribute to the observed higher incidence in these groups.

When comparing internationally, incidence rates in the Netherlands were lower than those reported in other high-income countries: the UK (57 per 100,000 PYAR),^14^ North-America (47–77 per 100,000 PYAR in those >45 years),^15^ and Taiwan (36.6 per 100,000 PYAR in those >40 years).^5^ These differences may reflect, however, variations in study design, population coverage, case ascertainment methodologies, and environmental exposures, including the phase-out of toxins such as paraquat and trichloroethylene.

Within our study period, a stable incidence from 2017 to 2019 was followed by a notable decline in 2020, a slight rebound in 2021, and another decrease in 2022. The decrease in 2020 is likely attributable to delayed diagnoses resulting from healthcare disruptions caused by the COVID-19 pandemic, and was followed by a partial compensatory increase in 2021. In the Netherlands, delays in diagnoses of several types of cancer,^43^ screening programmes (for e.g., hip dysplasia),^44^ and other diagnostic procedures (for e.g., gastroenteric diseases)^45^ have been reported for 2020. While delayed PD diagnoses may have affected temporal incidence estimates, it is unlikely that they substantially distorted the spatial patterns. Such distortion would require differential delays in diagnosis across regions or hospitals, which is not expected given the relatively uniform access to neurological care in the Netherlands. The incidence during the pre-pandemic period (2017–2019) was stable. While this three-year window is too short to robustly evaluate long-term temporal trends, it is indicative that major temporal changes were not occurring immediately before the pandemic. Furthermore, a better recognition of atypical parkinsonism by neurologists in the last years may have led to an artefactual decrease in PD; this notion may explain why the prevalence of atypical parkinsonism is growing faster than that of PD in the Netherlands.^46^ The unavailability of DHD data from 2023 likely contributed to a slight underestimation of PD cases, predominantly in 2022. Thus, the decline from 2019 to 2022 and the ∼12% reduction in 2017–2022 may not reflect a true reduction in PD incidence but rather pandemic-related diagnostic delays.

Misclassification of PD cases remains a concern, potentially leading to both under- and overestimation of incidence rates. While hospital records capture PD-related admissions well, they may prioritize the primary reason for admission, possibly missing cases where PD was not the primary diagnosis (e.g., fractures). Health insurance data may underestimate incidence among the oldest age groups (>90 years), where comorbidities such as dementia or osteoporosis could obscure PD symptoms. Furthermore, because this data source relies heavily on hospital data, PD cases from persons living in assisted living facilities can be missed, contributing to the underestimation of PD incidence. The higher number of PD cases identified in 2016 (n = 4534) raised concerns about potential inclusion of prevalent cases, as individuals starting medication after 2016 could not be reliably classified as incident cases. We therefore restricted the study period to 2017–2022. Excluding cases with uncertain diagnosis years likely led to a 5–10% underestimation of total PD incidence. Overdiagnosis may result from the presence of other neurodegenerative diseases or parkinsonism that have not (yet) been clearly distinguished from PD. By following individuals prospectively, we minimized misclassification of parkinsonism as PD cases. We identified a 5% diagnosis conversion rate from PD to parkinsonism within 1–2 years after diagnosis, which is within the 0–25% conversion rates reported by Rizzo and colleagues.^47^ Our sensitivity analysis restricted to 2017–2019 supported the observed IR for the whole period. The use of multiple data sources reduced potential over- and underestimation of cases arising from the inherent limitations of individual data sources. However, due to strict privacy regulations governing these databases, re-identification of individuals is not permitted. Consequently, further linkage with general practitioner records or active follow-up with patients or next of kin was not possible. Therefore, some degree of misclassification bias cannot be entirely excluded.

This comprehensive assessment provides valuable insights into PD incidence trends in the Netherlands, highlighting stable overall incidence but notable demographic and geographic disparities. The PD case ascertainment strategy developed in this study offers a robust tool for future research using administrative health data and creates new opportunities for investigating environmental contributors, such as pesticide exposure, using individual-level data.

## Contributors

All authors (MS, SP, SH, SD, BB, RV) contributed to the study conceptualisation and design, the development of the algorithm for case ascertainment and interpretation of the results. MS undertook the linkage of the several data sources to create the study population, verified the data, conducted the statistical analyses, generated figures and tables, and drafted the first version of the manuscript. SP, AH and RV supervised all stages of the study. RV and BB acquired funding. RV was responsible for project administration. MS and RV directly accessed the raw data. All authors critically reviewed and edited the manuscript, and approved the final version. RV had final responsibility for the decision to submit for publication. Throughout all stages of the study, including drafting the manuscript, no artificial intelligence tools were used.

## Data sharing statement

All data used in this study is available as non-public microdata in the remote secure environment of Statistics Netherlands (CBS) [project number 9234, Utrecht University]. Under certain conditions, these microdata are accessible for statistical and scientific research. For further information: https://www.cbs.nl/en-gb/our-services/customised-services-microdata/microdata-conducting-your-own-research.

## Editor note

The Lancet Group takes a neutral position with respect to territorial claims in published maps and institutional affiliations.

## Declaration of interests

Prof. Bastiaan R. Bloem serves as the co-Editor in Chief for the Journal of Parkinson's disease, serves on the editorial board of Digital Biomarkers, is a member of Movement Disorder Society, the Critical Path Institute for Parkinson and the KNAW Van Leersum Fund Review Committee, has received fees from serving on the scientific advisory board for the Critical Path Institute, Gyenno Science, MedRhythms, UCB, Kyowa Kirin and Zambon (paid to the Institute), has received fees for speaking at conferences from AbbVie, Bial, Biogen, GE Healthcare, Oruen, Roche, UCB and Zambon (paid to the Institute), and has received research support from Biogen, Cure Parkinson's, Davis Phinney Foundation, Edmond J. Safra Foundation, Fred Foundation, Gatsby Foundation, Hersenstichting Nederland, Horizon 2020, IRLAB Therapeutics, Maag Lever Darm Stichting, Michael J Fox Foundation, Ministry of Agriculture, Ministry of Economic Affairs & Climate Policy, Ministry of Health, Welfare and Sport, Netherlands Organization for Scientific Research (ZonMw), Not Impossible, Parkinson Vereniging, Parkinson's Foundation, Parkinson's UK, Stichting Alkemade-Keuls, Stichting Parkinson NL, Stichting Woelse Waard, Health Holland/Topsector Life Sciences and Health, UCB, Verily Life Sciences, Roche and Zambon. Prof. Bloem does not hold any stocks or stock options with any companies that are connected to Parkinson's disease or to any of his clinical or research activities.

Dr. Sirwan K. L. Darweesh serves as co-chair of the Movement Disorders Society Epidemiology Study Group, has received fees for speaking at conferences and podcasts from AbbVie (paid to the institute), Parkinson's Foundation, ParkinsonNet and the Belgian Neurological Society, and has received research support from the Parkinson's Foundation, ParkinsonNL, Michael J Fox Foundation, ERA4Health, Gieskes Strijbis Foundation, the Netherlands Organisation for Health and Edmond J Safra Foundation.

The remaining authors declare no competing interests.
