# Supplementary material for: Incidence and spatial variation of Parkinson's disease in the Netherlands (2017–2022): a population-based study
Source: Lancet Reg Health Eur. 2026 Jan 21;62:101565. doi: 10.1016/j.lanepe.2025.101565 (PMC12959304; doi:10.1016/j.lanepe.2025.101565)
Supplement: Supplementary Material [file mmc1.docx]

**Supplementary material**

**Table of contents**

[Table S.1 – Data sets from Statistics Netherlands (CBS) used in the study. 2](#_Toc212044186)

[Figure S.1 – Overview of key datasets used datasets and their linkability via unique identifiers. 3](#_Toc212044187)

[Textbox 1 – Further details on the on the Bayesian hierarchical model 3](#_Toc212044188)

[Table S.2 – Sensitivity analyses using different spatial weights when computing Global Moran’s I 3](#_Toc212044189)

[Table S.3 – Number of persons that obtained score 1, 2 and 3 after application of the algorithm to identify PD cases. 4](#_Toc212044190)

[Table S.5 – Overall yearly Incidence Rate (IR) of Parkinson’s Disease per 100,000 person-years-at-risk. 4](#_Toc212044191)

[Table S.6 – Incidence Rate (IR) of Parkinson’s Disease per 100,000 person-years-at-risk in 2017-2022 per sex and age group and corresponding male-to-female ratios. 4](#_Toc212044192)

[Table S.7 – Age and sex standardized incidence Rate (IR) of Parkinson’s Disease per 100,000 person-years-at-risk in 2017-2022 from sensitivity analyses. 5](#_Toc212044193)

[Table S.9 – Age standardized incidence Rate (IR) of Parkinson’s Disease per 100,000 person-years-at-risk in 2017-2022, stratified by Sex & Migration background and by Sex & Province. 6](#_Toc212044194)

[Figure S.2 – Risk ratios of Parkinson’s Disease by Neighborhood in the Netherlands (2017–2022) in (a) the male population, (b) the female population, (c) the 60-80 year-old population, and (d) the population over 80 years of age. For some neighborhoods, the total number of cases for the computation of the smoothed RRs was less than 10 and are therefore not reported (NA). 7](#_Toc212044195)

[Figure S.3 – Cluster analysis on the Rate Ratios (RR) of PD incidence computed per neighborhood. Striped areas correspond to areas where the average RR is >1. The number of clusters was set from 4 to 10 in increments of 2 to explore the effect of choice of cluster numbers on the interpretation of the results. 9](#_Toc212044196)

[References 10](#_Toc212044197)

# Table S.1 – Data sets from Statistics Netherlands (CBS) used in the study.

| **Data set name (Dutch)** | **Description** | **Variables used** | **Definition** |
| --- | --- | --- | --- |
| GBAPERSOON | Personal characteristics of persons in the Personal Records Database (BRP) | RINPERSOON  GBAGEBOORTELAND  GBAGESLACHT  GBAGEBOORTEJAAR  GBAGEBOORTEMAAND  GBAGEBOORTEDAG | *Pseudoanonimyzed identifier of a person*  Country of birth  Sex  Year of birth  Month of birth  Day of birth |
| GBAADRESOBJECTBUS | Address characteristics of persons registered in the BRP | RINPERSOON  RINOBJECTNUMMER  GBADATUMAANVANGADRESHOUDING  GBADATUMEINDEADRESHOUDING | *Pseudoanonimyzed identifier of a person*  *Pseudoanonimyzed identifier of an address of a person*  Start date of residence at address  End date of residence at address |
| GBAOVERLIJDENTAB | Date of death of persons registered in the BRP | RINPERSOON  GBADatumOverlijden | *Pseudoanonimyzed identifier of a person*  Date of death |
| DOODOORZTAB | Causes of death | RINPERSOON  UCCODE | *Pseudoanonimyzed identifier of a person*  Underlying cause of death (ICD-10) |
| MEDICIJNTAB | Medications by ATC code (4 positions) | RINPERSOON  ATC4 | *Pseudoanonimyzed identifier of a person*  Classification of medication groups based on 4-position ATC (Anatomical, Therapeutic, Chemical) code |
| MSZPrestatiesVEKTTAB | Opened Diagnosis Treatment Combination (DBC) procedures in Specialist Medical Care (Vetkis) | RINPERSOON  VEKTMSZBegindatumPrest  VEKTMSZEinddatumPrest  VEKTMSZSpecialismeDiagnoseCombinatie | *Pseudoanonimyzed identifier of a person*  Start date of healthcare procedure  End date of healthcare procedure  Specialty-specific diagnosis for the care procedure |
| LMR_BASIS | National Medical Registration | RINPERSOON  Opndat  hfddiag | *Pseudoanonimyzed identifier of a person*  Start date of hospitalization  Main diagnosis according to ICD10 |
| LBZBASISTAB | National Basic Hospital Care Register | RINPERSOON  LBZOpnamedatum  LBZIcd10hoofddianose | *Pseudoanonimyzed identifier of a person*  Start date of hospitalization  Main diagnosis according to ICD10 |
| PARTNERBUS | Individuals with a cohabiting partner | RINPERSOON  AANVANGPARTNER  EINDEPARTNER | *Pseudoanonimyzed identifier of a person*  Start date of cohabitation  End date of cohabitation |
| KOPPELPERSOONHUISHOUDEN | Linkage between main incomer and household members | RINPERSOON  RINPERSOONHKW | *Pseudoanonimyzed identifier of a person*  *Pseudoanonimyzed identifier of the main income earner of the household* |
| INHATABV | Household income | RINPERSOONHKW  INHP100HBEST | *Pseudoanonimyzed identifier of the main income earner of the household*  Percentile groups of disposable household income |
| SESWOA | Household socioeconomic status scores | RINPERSOONHKW  TOTAALSCORE | *Pseudoanonimyzed identifier of the main income earner of the household*  Overall household socioeconomic status score based on financial wealth, educational level, and recent employment history |
| VSLGWBTAB | Municipality, neighborhood and sub-neighborhood codes for residential addresses | RINOBJECTNUMMER  WCJJJJ | *Pseudoanonimyzed identifier of an address of a person*  *Neighborhood code* |
| Wijk_en_Buurtstatistieken/kwb | Municipality, neighborhood and sub-neighborhood characteristics | gwb_code  ste_mvs | Municipality code, *neighborhood code* and sub-neighborhood code  Urbanization degree |
| Wijk_en_Buurtstatistieken/SES-WOA | Municipality, neighborhood and sub-neighborhood socioeconomic characteristics | WijkenEnBuurten  SESWOATotaalscoreGemiddelscore | Municipality code, *neighborhood code* and sub-neighborhood code  Socioeconomic status score based on financial wealth, educational level, and recent employment history from households in the neighborhood |

# Figure S.1 – Overview of key datasets used datasets and their linkability via unique identifiers.

Potential linkage to environmental exposures via residential address is also depicted.

DHD = Dutch Hospital Data; Vektis = health insurance data on provided healthcare services; SEP = socio-economic position


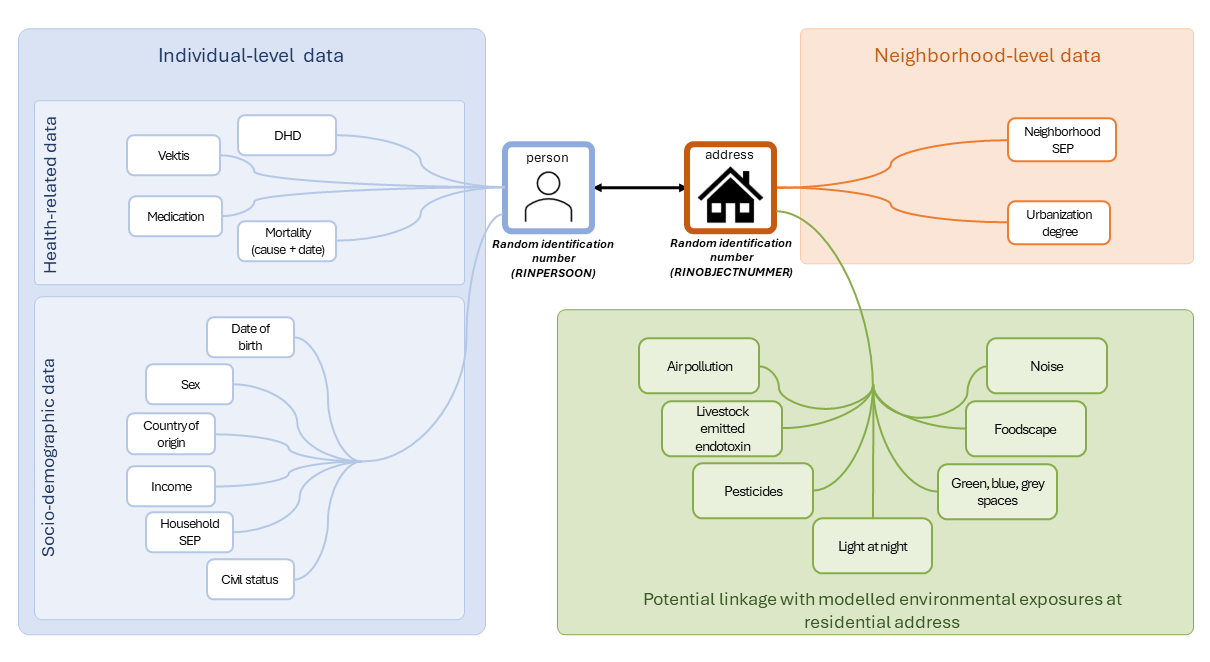


**Textbox 1 – Further details on the on the Bayesian hierarchical model**

We modelled area-level PD counts using a Bayesian hierarchical Poisson model implemented with the integrated nested Laplace approximation (INLA) as the method framework for efficient Bayesian inference, following the approach of Moraga (2020).^1^ The number of observed cases in each of the 2,980 neighbourhoods in the Netherlands was compared to the number of expected cases based on the population structure (specifically, an internal reference population stratified by sex and 5-year age groups) to estimate relative risks (RR). The model incorporated two random effects: one capturing spatially structured variation (which assumed similar risks in neighbouring areas) and another capturing unstructured random variation (differences which are not related to location). The structured component followed a Conditional Autoregressive (CAR) prior (Besag model) based on shared boundaries between neighbourhoods, while the unstructured component followed an independent and identically distributed (i.i.d.) Gaussian prior, which are the default priors in the INLA R package. The degree of spatial smoothing was controlled by the estimated precision parameters of the structured and unstructured random effects, which were automatically inferred from the data. The precision parameters were assigned Penalized Complexity (PC) priors, also as implemented by default in the INLA R package. These specifications correspond to the Besag–York–Mollié (BYM) spatial model, which smooths disease risk estimates by borrowing information from neighbouring areas, producing more stable estimates in regions with small populations or few cases.

# Textbox 1 – Further details on the on the Bayesian hierarchical model

We modelled area-level PD counts using a Bayesian hierarchical Poisson model implemented with the integrated nested Laplace approximation (INLA) as the method framework for efficient Bayesian inference, following the approach of Moraga (2020).^1^ The number of observed cases in each of the 2,980 neighbourhoods in the Netherlands was compared to the number expected cases based on the population structure (specifically, an internal reference population stratified by sex and 5-year age groups) to estimate relative risks (RR). The model incorporated two random effects: one capturing spatially structured variation (which assumed similar risks in neighbouring areas) and another capturing unstructured random variation (differences which are not related to location). The structured component followed a Conditional Autoregressive (CAR) prior (Besag model) based on shared boundaries between neighbourhoods, while the unstructured component followed an independent and identically distributed (i.i.d.) Gaussian prior, which are the default priors in the INLA R package. The degree of spatial smoothing was controlled by the estimated precision parameters of the structured and unstructured random effects, which were automatically inferred from the data. The precision parameters were assigned Penalized Complexity (PC) priors, also as implemented by default in the INLA R package. These specifications correspond to the Besag–York–Mollié (BYM) spatial model, which smooths disease risk estimates by borrowing information from neighbouring areas, producing more stable estimates in regions with small populations or few cases.

#

# Table S.2 – Sensitivity analyses using different spatial weights when computing Global Moran’s I

| **Spatial weights** | **Analysis** | **Global Moran's I statistic** | **P value** |
| --- | --- | --- | --- |
| Contiguity + Euclidean distance for the costs of edges | main | 0·83 (0·81, 0·86) | <0·0001 |
| Contiguity only | sensitivity | 0·87 (0·85, 0·89) | <0·0001 |
| Euclidean distance | sensitivity | 0·84 (0·82, 0·87) | <0·0001 |
| k-nearest neighbour | sensitivity | 0·95 (0·93, 0·96) | <0·0001 |

# Table S.3 – Number of persons that obtained score 1, 2 and 3 after application of the algorithm to identify PD cases.

| **Data source combination** | **PD score** | **N** | **Analysis** |
| --- | --- | --- | --- |
| medication data | score 1 | 79278 | not PD case |
| DHD | score 2 | 209 | excluded |
| DHD, mortality registry |  | 16 |  |
| DHD, mortality registry, Vektis |  | 25 |  |
| mortality registry, Vektis |  | 54 |  |
| Vektis |  | 1941 |  |
| DHD, medication data | score 3 | 358 | PD case |
| DHD, medication data, mortality registry |  | 65 |  |
| DHD, medication data, mortality registry, Vektis |  | 134 |  |
| DHD, medication data, Vektis |  | 640 |  |
| medication data, mortality registry |  | 943 |  |
| medication data, mortality registry, Vektis |  | 987 |  |
| medication data, Vektis |  | 19216 |  |
| mortality registry | n/a | 844 | excluded |

#

# Table S.5 – Overall yearly Incidence Rate (IR) of Parkinson’s Disease per 100,000 person-years-at-risk.

|  | **Incidence Rate (95% CI) per 100,000 person-years at-risk** | | | | | | |
| --- | --- | --- | --- | --- | --- | --- | --- |
|  | **2017-2022** | **2017** | **2018** | **2019** | **2020** | **2021** | **2022** |
| crude | 21·3 (21·0, 21·6) | 21·8 (21·1, 22·5) | 22·5 (21·8, 23·2) | 22·2 (21·5, 22·9) | 19·8 (19·1, 20·5) | 21·7 (21·0, 22·3) | 20·8 (20·1, 21·4) |
| sex and 5-year age groups standardized | 21·8 (21·6, 22·1) | 22·6 (21·9, 23·3) | 22·9 (22·2, 23·6) | 22·2 (21·5, 22·9) | 19·5 (18·8, 20·1) | 20·9 (20·2, 21·6) | 19·8 (19·2, 20·5) |

# Table S.6 – Incidence Rate (IR) of Parkinson’s Disease per 100,000 person-years-at-risk in 2017-2022 per sex and age group and corresponding male-to-female ratios.

| **Age** | **Sex** | **Incidence Rate (95% CI) per 100,000 person-years at-risk^1^** | **Male-to-female ratio** |
| --- | --- | --- | --- |
| <40 | Male | 0·1 (0·1, 0·1) | 2·22 |
|  | Female | 0·0 (0·0, 0·1) |  |
| 40-50 | Male | 3·5 (3·0, 3·9) | 1·66 |
|  | Female | 2·1 (1·7, 2·4) |  |
| 51-60 | Male | 16·3 (15·4, 17·2) | 2·04 |
|  | Female | 8·0 (7·3, 8·6) |  |
| 61-70 | Male | 53·7 (51·9, 55·6) | 1·93 |
|  | Female | 27·9 (26·6, 29·2) |  |
| 71-80 | Male | 134·8 (131·4, 138·1) | 1·76 |
|  | Female | 76·8 (74·3, 79·2) |  |
| 81-90 | Male | 150·3 (144·4, 156·1) | 1·61 |
|  | Female | 93·3 (89·4, 97·2) |  |
| >90 | Male | 35·3 (27·6, 42·9) | 1·74 |
|  | Female | 20·3 (16·6, 23·9) |  |

^1^ 5-year age groups standardized

# Table S.7 – Age and sex standardized incidence Rate (IR) of Parkinson’s Disease per 100,000 person-years-at-risk in 2017-2022 from sensitivity analyses.

|  |  | **Main analysis** | **Restriction to individuals with at least three years of follow-up** | **Censoring individuals upon entry into a nursing home or mental health institution** |
| --- | --- | --- | --- | --- |
| **Overall** | crude | 21·3 (21·0, 21·6) | 21·9 (21·5, 22·3) | 21·6 (21·3, 21·8) |
|  | sex- and age-standardized | 21·8 (21·6, 22·1) | 22·5 (22·1, 22·9) | 22·7 (22·5, 23·0) |

**Table S.8 – Sex standardized incidence Rate (IR) of Parkinson’s Disease per 100,000 person-years-at-risk in 2017-2022, stratified by Age & Province.**

| **Province** | **40-50** | **51-60** | **61-70** | **71-80** | **81-90** | **>90** |
| --- | --- | --- | --- | --- | --- | --- |
| Groningen | 2·5 (1·1, 3·8) | 12·3 (9·3, 15·2) | 47·1 (40·7, 53·5) | 114·0 (102·4, 125·6) | 139·7 (119·9, 159·4) |  |
| Friesland | 4·1 (2·4, 5·8) | 11·6 (8·8, 14·5) | 41·9 (36·1, 47·6) | 112·7 (101·9, 123·5) | 133·7 (116·0, 151·5) | 49·0 (25·1, 73·0) |
| Drenthe | 3·4 (1·6, 5·3) | 18·3 (14·2, 22·4) | 38·5 (32·3, 44·8) | 102·5 (90·9, 114·2) | 126·8 (107·5, 146·0) | 39·5 (15·8, 63·3) |
| Overijssel | 2·8 (1·7, 3·8) | 12·2 (10·1, 14·4) | 36·2 (32·0, 40·3) | 95·1 (87·4, 102·9) | 126·5 (113·1, 139·8) | 18·7 (7·1, 30·3) |
| Flevoland |  | 14·1 (9·5, 18·6) | 35·7 (28·2, 43·2) | 90·3 (74·2, 106·5) | 143·9 (109·2, 178·6) |  |
| Gelderland | 2·6 (1·8, 3·3) | 11·9 (10·3, 13·5) | 43·4 (40·1, 46·7) | 101·2 (95·4, 107·1) | 121·8 (112·0, 131·5) | 43·2 (30·2, 56·3) |
| Utrecht | 4·2 (2·9, 5·5) | 11·0 (9·1, 13·0) | 41·6 (37·4, 45·8) | 108·4 (100·4, 116·5) | 135·1 (121·2, 149·0) | 55·4 (35·6, 75·2) |
| Noord-Holland | 2·4 (1·8, 3·1) | 12·0 (10·7, 13·4) | 40·7 (37·9, 43·5) | 105·0 (99·8, 110·2) | 139·5 (130·3, 148·7) | 52·7 (40·1, 65·2) |
| Zuid-Holland | 3·2 (2·6, 3·9) | 12·1 (11·0, 13·3) | 40·7 (38·3, 43·1) | 106·2 (101·6, 110·8) | 127·2 (119·6, 134·8) | 27·4 (19·7, 35·1) |
| Zeeland |  | 10·2 (6·7, 13·7) | 29·2 (23·0, 35·5) | 88·5 (76·4, 100·6) | 70·0 (54·2, 85·8) |  |
| Noord-Brabant | 2·3 (1·7, 3·0) | 12·6 (11·1, 14·1) | 39·2 (36·4, 42·0) | 99·4 (94·3, 104·6) | 122·6 (114·0, 131·3) | 38·0 (26·5, 49·6) |
| Limburg | 2·5 (1·4, 3·5) | 10·3 (8·4, 12·2) | 36·6 (32·8, 40·4) | 88·0 (81·1, 94·9) | 115·1 (103·5, 126·6) | 25·2 (12·6, 37·8) |

Blank cells correspond to IRs that were not calculated due to low number of cases (<10).

# Table S.9 – Age standardized incidence Rate (IR) of Parkinson’s Disease per 100,000 person-years-at-risk in 2017-2022, stratified by Sex & Migration background and by Sex & Province.

|  |  | **males** | **females** | **male-to-female ratio** |
| --- | --- | --- | --- | --- |
| **Migration background** | The Netherlands | 28·4 (27·9, 28·9) | 15·8 (15·4, 16·2) | 1·80 |
|  | Morocco | 34·9 (31·6, 38·1) | 25·2 (22·4, 28·0) | 1·38 |
|  | Turkey | 27·6 (24·7, 30·4) | 18·9 (16·5, 21·3) | 1·46 |
|  | Suriname | 24·6 (21·6, 27·6) | 14·0 (11·9, 16·2) | 1·75 |
|  | Former Dutch Antilles and Aruba | 30·0 (25·3, 34·6) | 15·8 (12·4, 19·2) | 1·46 |
|  | Other non-Western countries | 25·4 (23·7, 27·2) | 13·8 (12·8, 14·8) | 1·73 |
|  | Other Western countries | 27·2 (25·8, 28·6) | 14·7 (13·4, 16·1) | 1·97 |
| **Province** | Groningen | 31·4 (28·9, 33·9) | 17·1 (15·3, 19·0) | 1·83 |
|  | Friesland | 29·9 (27·5, 32·3) | 17·2 (15·4, 19·1) | 1·73 |
|  | Drenthe | 30·5 (27·7, 33·3) | 15·1 (13·2, 17·1) | 2·02 |
|  | Overijssel | 26·2 (24·5, 27·8) | 15·1 (13·8, 16·4) | 1·74 |
|  | Flevoland | 30·8 (27·5, 34·1) | 12·0 (10·0, 14·1) | 2·56 |
|  | Gelderland | 28·3 (27·0, 29·6) | 15·7 (14·7, 16·7) | 1·80 |
|  | Utrecht | 29·3 (27·6, 31·0) | 17·1 (15·8, 18·4) | 1·71 |
|  | Noord-Holland | 29·3 (28·1, 30·4) | 16·4 (15·5, 17·2) | 1·79 |
|  | Zuid-Holland | 28·5 (27·5, 29·5) | 16·2 (15·5, 17·0) | 1·75 |
|  | Zeeland | 23·5 (20·7, 26·2) | 9·2 (7·4, 10·9) | 2·56 |
|  | Noord-Brabant | 27·7 (26·5, 28·9) | 15·1 (14·2, 16·0) | 1·83 |
|  | Limburg | 24·9 (23·2, 26·5) | 13·5 (12·2, 14·7) | 1·85 |

#

# Figure S.2 – Risk ratios of Parkinson’s Disease by Neighbourhood in the Netherlands (2017–2022) in (a) the male population, (b) the female population, (c) the 60-80 year-old population, and (d) the population over 80 years of age. For some neighbourhoods, the total number of cases for the computation of the smoothed RRs was less than 10 and are therefore not reported (NA).

| **(a)** | 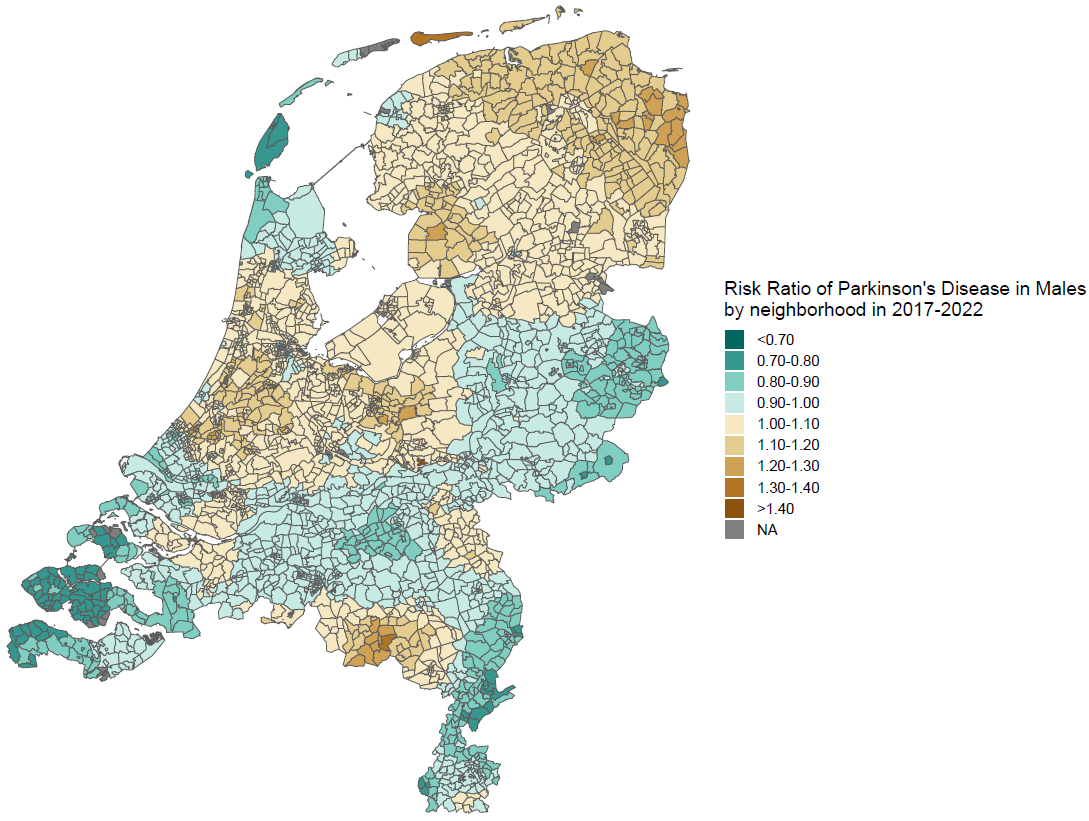 |
| --- | --- |
|  |  |
| **(b)** | 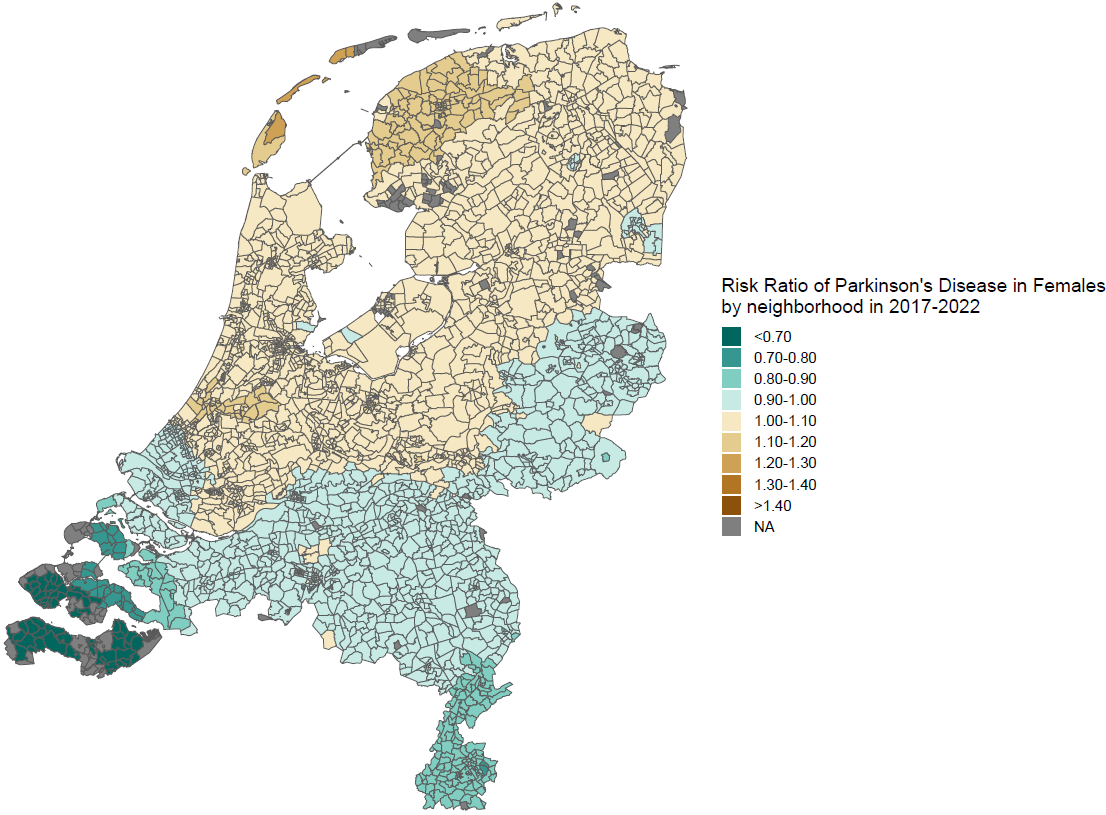 |
|  |  |
| **(c)** | 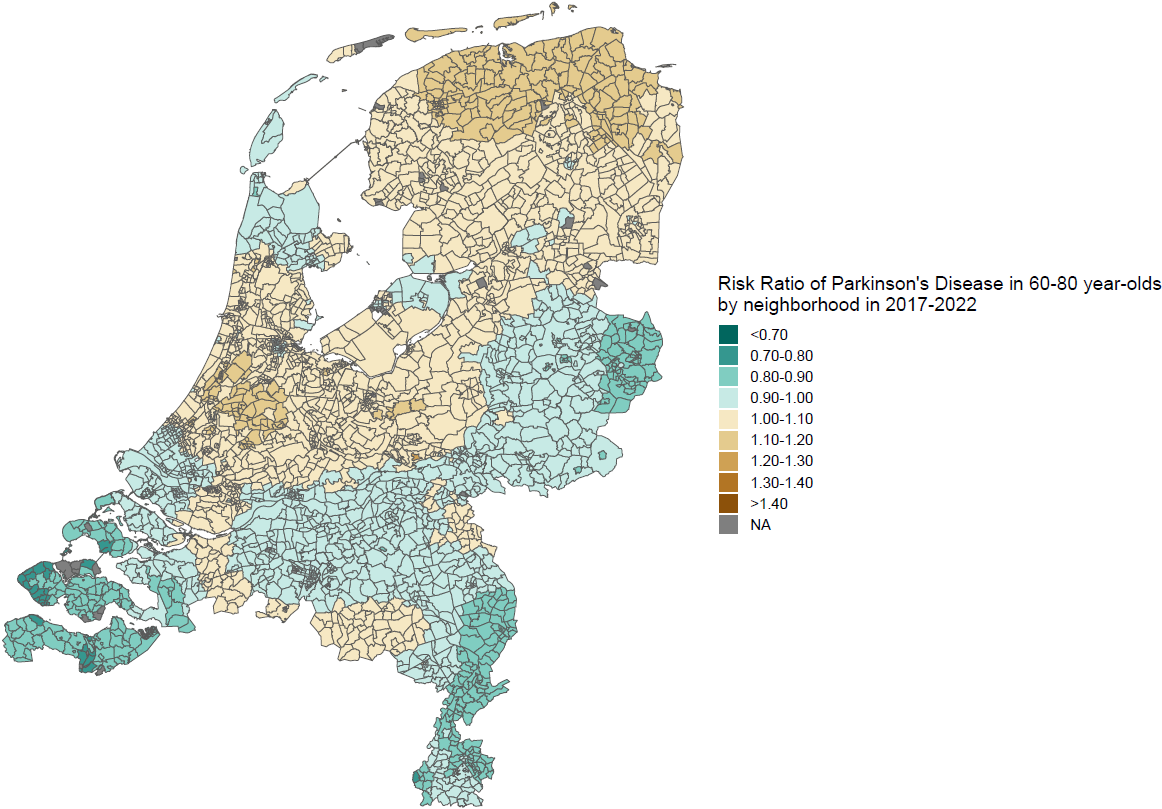 |
|  |  |
| **(d)** | 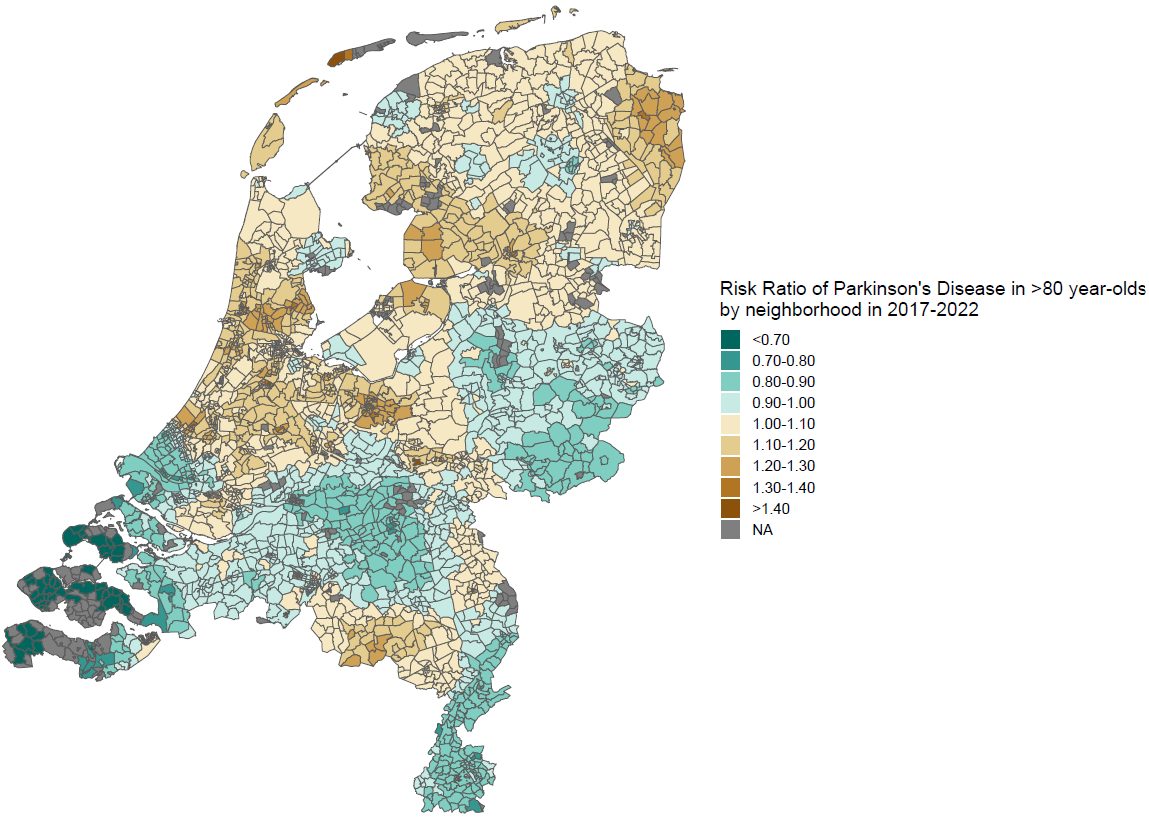 |

# Figure S.3 – Cluster analysis on the Rate Ratios (RR) of PD incidence computed per neighbourhood. Striped areas correspond to areas where the average RR is >1. The number of clusters was set from 4 to 10 in increments of 2 to explore the effect of choice of cluster numbers on the interpretation of the results.

| 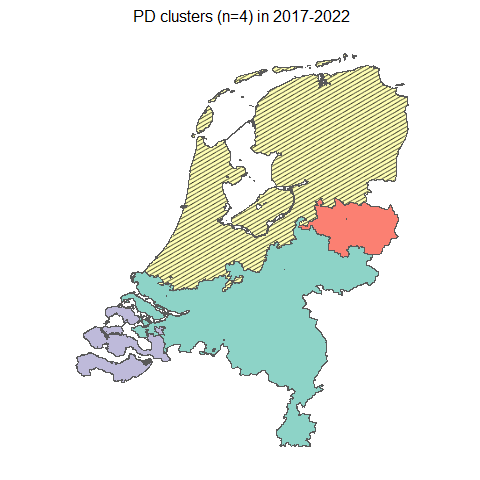 | 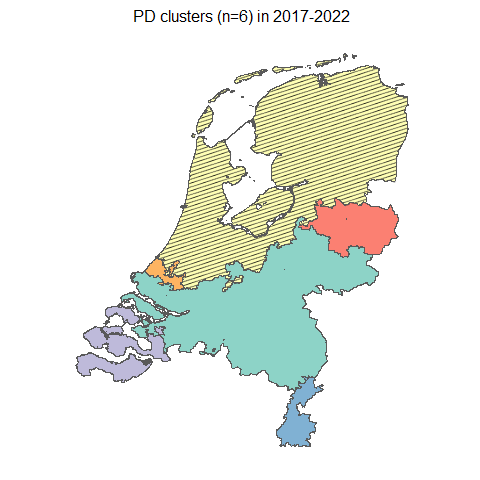 |
| --- | --- |
| 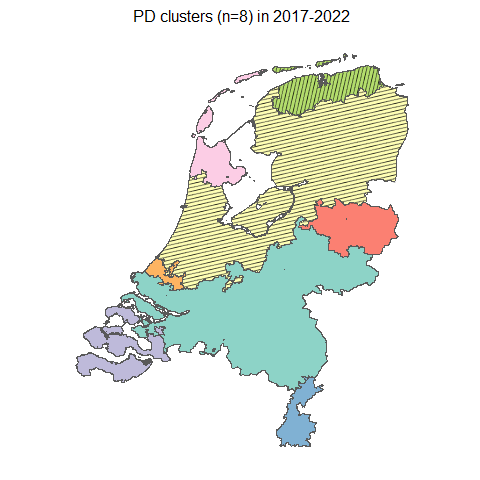 | 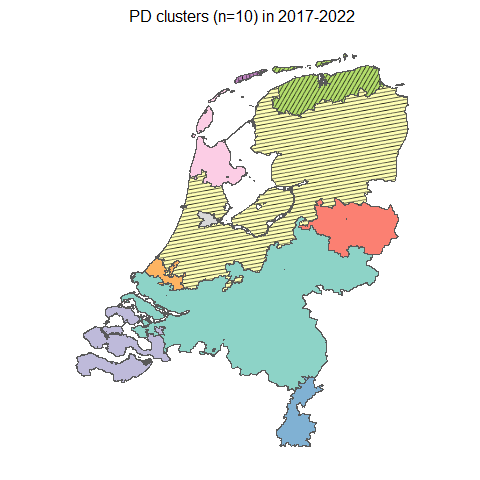 |

# References

1. Moraga P. Spatial Statistics for Data Science: Theory and Practice with R: Chapman & Hall; 2023.
